# [Retracted] Effect of CCL2 siRNA on proliferation and apoptosis in the U251 human glioma cell line

**DOI:** 10.3892/mmr.2023.13124

**Published:** 2023-11-06

**Authors:** Bin Lu, Yue Zhou, Zhongzhou Su, Ai Yan, Peng Ding

Mol Med Rep 16: 3387–3394, 2017; DOI: 10.3892/mmr.2017.6995

Following the publication of this paper, it was drawn to the Editor's attention by a concerned reader that the VEGF western blotting data shown in Fig. 4A on p. 3392 were strikingly similar to data appearing in different form in other articles written by different authors at different research institutes.

Owing to the fact that the contentious data in the above article had already been published elsewhere prior to its submission to *Molecular Medicine Reports*, the Editor has decided that this paper should be retracted from the Journal. The authors were asked for an explanation to account for these concerns, but the Editorial Office never received a reply. The Editor apologizes to the readership for any inconvenience caused.

